# The effect of high-frequency rTMS of the left dorsolateral prefrontal cortex on the resolution of response, semantic and task conflict in the colour-word Stroop task

**DOI:** 10.1007/s00429-021-02237-4

**Published:** 2021-02-19

**Authors:** Benjamin A. Parris, Michael G. Wadsley, Gizem Arabaci, Nabil Hasshim, Maria Augustinova, Ludovic Ferrand

**Affiliations:** 1 Department of Psychology, Bournemouth University, Talbot Campus, Fern Barrow, Poole, Dorset, BH12 5BB UK; 2 School of Applied Sciences, De Montfort University, Leicester, UK; 3 Normandie Université, UNIROUEN, CRFDP, Rouen, France; 4 Université Clermont Auvergne, CNRS LAPSCO, Clermont-Ferrand, France

**Keywords:** TMS, Dorsolateral prefrontal cortex, Stroop, Cognitive control, Selective attention

## Abstract

Previous work investigating the effect of rTMS of left Dorso-Lateral Prefrontal Cortex (DLPFC) on Stroop task performance reports no changes to the Stroop effect but reduced reaction times on both congruent and incongruent trials relative to sham stimulation; an effect attributed to an enhanced attentional (or task) set for colour classification. The present study tested this account by investigating whether, relative to vertex stimulation, rTMS of the left DLPFC modifies task conflict, a form of conflict that arises when task sets for colour classification and word reading compete, given that this particular type of conflict would be reduced by an enhanced task set for colour classification. Furthermore, the present study included measures of other forms of conflict present in the Stroop task (response and semantic conflict), the potential effects on which would have been hidden in previous studies employing only incongruent and congruent stimuli. Our data showed that left DLPFC stimulation had no effect on the magnitude of task conflict, nor did it affect response, semantic or overall conflict (where the null is supported by sensitive Bayes Factors in most cases). However, consistent with previous research left DLPFC stimulation had the general effect of reducing reaction times. We, therefore, show for the first time that relative to real vertex stimulation left DLPFC stimulation does not modify Stroop interference. Alternative accounts of the role of the left DLPFC in Stroop task performance in which it either modifies response thresholds or facilitates responding by keeping the correct response keys active in working memory are discussed.

## Introduction

Since its inception (Stroop 1935), the Stroop task has become an important clinical tool and has been referred to as the gold-standard measure of selective attention (MacLeod 1992). The Stroop interference effect is the robust finding that people are slower to name a colour that a word is printed in when the word spells out a different colour (i.e., incongruent trials- the word ‘red’ printed in green) compared to a baseline compound stimulus (i.e., colour-neutral trials- the word ‘top’/ string of xxx printed in red). It is often viewed as a unitary phenomenon that results entirely from the single-stage of response competition that colour-incongruent trials produce (see e.g., MacLeod 1992 for a review).

Yet, abundant research suggests that interference in the Stroop task is potentially underpinned by conflict at various points in the processing stream including at the level of task set activation, semantic activation and response output (Klein 1964; Monsell et al. 2001; see Parris et al. (under review)). In this view, *task conflict* derives from the simultaneous preparation of two task sets (word reading vs. colour naming; Goldfarb and Henik 2007; Parris 2014) which creates conflict even before the identity of the Stroop stimulus has been revealed (Hershman and Henik 2019). Subsequent processing of the meaning of the word results in *semantic conflict* when the irrelevant word dimension gives conflicting information about which is the target stimulus (Augustinova and Ferrand 2012, 2014; Augustinova et al. 2015,2018; White et al. 2016). Finally, when the irrelevant word is also a possible response option (i.e., incongruent trials) it creates conflict at the response level (i.e., *response conflict*, Hasshim and Parris 2018; Milham et al. 2001).

### The neural mechanisms of Stroop task performance

The specific neural mechanisms involved in guiding our attention in the Stroop task still remain a source of debate. The dorsolateral prefrontal cortex (DLPFC), along with the anterior cingulate cortex (ACC), has long been considered central to the circuitry of attentional control (MacDonald et al. 2000). Popular models of selective attention posit that the ACC is responsible for detecting the presence of response conflict between competing representations and consequently engages the DLPFC to impose cognitive control (e.g., Banich 2009, 2019; Banich et al. 2019; Botvinick et al. 2001; MacDonald et al. 2000). This theory is supported by neuroimaging studies that link selective attention in the Stroop task to activity in these regions and in particular the left hemisphere of the brain (e.g., Adleman et al. 2002; Bench et al. 1993; Coderre et al. 2008; Khorram-Sefat et al. 1996; Langenecker et al. 2004; Liu et al. 2004; Mead et al. 2002; Parris et al. 2019a, b; Peterson et al. 1999; Song and Hakoda 2015; van Veen and Carter 2005; van Veen et al. 2001; Zysset et al. 2001). Whilst the role of both regions is a matter of ongoing debate (for debate around the function of the ACC see e.g. Aarts et al. 2008; Banich 2009, 2019; Botvinick et al. 2001; Khorram-Sefat et al. 1996; MacDonald et al. 2000; Mead et al. 2002; Milham et al. 2001; Parris et al. 2019a, b; Roelofs Van Turennout and Coles 2006; Song and Hakoda 2015; van Veen and Carter 2005; van Veen et al. 2001; Zysset et al. 2001), here we focus on the role of the left DLPFC.

A dissociation has been reported for the function of the DLPFC in a study considering the type of conflict presented. Van Veen and Carter (2005) reported that semantic conflict activated dorso-lateral prefrontal cortex (DLPFC: BA8/9), whereas response conflict activated more inferior lateral prefrontal cortex (BA9/44/45/46). In contrast, Milham and colleagues (Milham et al. 2001,2003) reported that both left and right PFC were activated by response conflict, but only left PFC was activated by semantic conflict. Consistently, a recent study by Parris et al. (2019a, b) reported that the left PFC plays an important role in the processing of both response and semantic conflict. However, since neuroimaging work is correlational it does not aid in the identification of the causal role of different brain regions in processing Stroop conflicts. One way to directly investigate the causal contribution of a neural region to attentional control is through the use of transcranial magnetic stimulation (TMS).

### Stimulation studies of the left DLPFC’s role in Stroop task performance

TMS is a non-invasive tool for modulating cortical activity (Wassermann et al. 2008). High frequency (≥ 5-Hz) repetitive TMS (rTMS) is typically considered to have an excitatory effect on underlying neurons, whereas the effects of low frequency (≤ 1-Hz) rTMS are considered inhibitory (Pell et al. 2011). Using rTMS to inhibit or facilitate brain regions thought to be responsible for attentional control, one could observe the effect on performance across different trial types and consequently determine whether specific regions are essential for resolving different types of conflict.

The few studies that have previously employed this technique to target the left DLPFC during Stroop task performance have reported that left DLPFC stimulation does not modify interference levels. Vanderhasselt et al. (2006) demonstrated that, compared to a sham condition, one session of high frequency (10-Hz) rTMS over the left DLPFC (location F3 on the international 10–20 EEG position system or BA8) decreased reaction times (RTs) to both congruent and incongruent trials. However, the stimulation did not modify the Stroop effect (incongruent – congruent trial RTs) and as such they concluded that their data were consistent with the notion that the role of the left DLPFC was to implement top-down attentional control by imposing a task set for colour classification.

In a more recent study, Li et al. (2017) investigated whether multiple sessions of rTMS of the left DLPFC (F3/B8) could improve Stroop task performance and explored the time course changes of cognitive processing after rTMS. Consistent with Vanderhasselt et al. (2006) they showed that compared to sham stimulation, rTMS reduced RTs to congruent and incongruent trials whilst making no difference to the Stroop interference effect indicating that left DLPFC plays no role in Stroop interference control; a finding that is also consistent with studies employing transcranial Direct Current Stimulation (Baumert et al. 2020; Fecteau et al. 2007, 2013; Loftus et al. 2015, although see Frings et al. 2018).

In the most recent assay investigating the effect of rTMS on Stroop task performance, Friehs et al. (2020) compared the effect of stimulation of the left DLPFC and right DLPFC to sham stimulation over the vertex. They showed that whilst right DLPFC stimulation resulted in the disruption of the congruency sequence effect (reduced Stroop interference on incongruent trials that follow incongruent trials in the presentation sequence), the left DLPFC did not modify Stroop interference nor the congruency sequence effect.

All of the above studies reported no significant reductions in Stroop interference following stimulation of the left DLPFC, but all three studies had important limitations: (1) neither Vanderhasselt et al. (2006) and Li et al. (2017) provided evidence for the null hypothesis of no effect on Stroop interference; evidence that can be provided by employing Bayes Factors (Dienes 2014, 2016; see for example Friehs et al. 2020); (2) neither Vanderhasselt et al. (2006) and Li et al. (2017) employed vertex stimulation as their baseline performance condition which better controls for the potential influence of somato-sensory effects on performance (Boschin, Mars and Buckley 2017; Duecker and Sack 2015; Hayward et al. 2004; Jung et al. 2016) and Friehs et al. (2020) used sham vertex stimulation where the TMS paddle is directed 45 degrees to the saggital plane and thus also results in no actual stimulation sensation; (3) the conclusions that can be drawn regarding the effect of left DLPFC stimulation on interference and/or conflict are also limited due to the use of only incongruent and congruent trials. Although these trial types are commonly employed to measure Stroop interference, it confounds the interfering effect of incongruent trials with facilitating effect of congruent items (MacLeod 1991; Parris et al. (under review)). Also, and importantly, it does not allow for the potential differential effect of left DLFPC stimulation on the different components of Stroop interference—task, semantic and response conflict. Given previous accounts of the role of the left DLPFC in Stroop task performance, task conflict, in particular, should be modified be left DLPFC stimulation.

### The present study

The aim of the present study was to investigate whether and the extent to which rTMS stimulation of left DLPFC affects overall, task, semantic and response conflict. Specifically, using a fully within-subjects design the present study set out to investigate whether facilitating left DLPFC region stimulated in previous studies (F3/BA8) differentially affects the resolution of these conflicts when compared to vertex stimulation and to use Bayes Factors to establish evidence for the null hypothesis of no difference. Given neuronavigation was unavailable we employed the Beam F3 approach to localisation (Beam et al. 2009).^1^ The Beam F3 method produces a reasonable approximation to neuronavigated localisation of left DLPFC (Mir-Moghtadaei et al. 2015) and has also been reported to be more precise and reliable than the 5.5 cm targeting method (Trapp et al. 2020). We initially aimed to test 20 participants but with the objective of collecting data until the Bayes Factor for the effect of stimulation on overall Stroop interference was sensitive (either for or against the null hypothesis).

To attribute the contribution of each conflict type to Stroop interference we ran a number of planned pairwise comparisons. For each comparison, we took a condition that permits the measurement of the relevant conflict type and compare this critical condition to a suitable baseline in which the relevant conflict is assumed to be absent (Hasshim and Parris 2014, 2015, 2018; Parris et al. 2019a, b). To index task conflict, we employed non-word letter strings (e.g., XXXX), which are thought to involve little to no conflict and compared them to non-colour-related neutral words (e.g., Kalanthroff et al. 2015; Monsell et al. 2001; Steinhauser and Hübner 2009). Semantic-associative trials (e.g., TOMATO, GRASS) are compared to neutral word trials as an index of semantic conflict and response conflict is isolated using an incongruent vs. non-response set comparison. Finally, overall interference is measured by comparing the incongruent condition against non-word letter strings condition. To maximise the distinction between conflict types in our experiment we employed a pure block trial type presentation method (Hasshim and Parris 2018).

Since task conflict results from a failure to fully engage the attentional set (Kalanthroff et al. 2018; Parris 2014), the litmus test for whether DLPFC is involved in setting attentional set, as others have argued, is the effect it has on the magnitude of task conflict. If the role of the left DLPFC in attentional control is to implement a general attentional or task set by keeping task-relevant information online (Botvinick et al. 2001; Li et al. 2017; Vanderhasselt et al. 2006), then enhancing DLPFC function using rTMS would increase the activation of the colour naming task set. Therefore, it would enable it to win out more easily over the competing endogenously activated task set for word reading. In short, enhancing DLPFC function using rTMS should reduce task conflict. If task conflict was indeed reduced, we would expect a reduction in RTs for all trials that involve this type of conflict (any trial with word stimuli: incongruent, non-response set, semantic associative and neutral word trials). Since repeated letter trials do not include irrelevant words, there would be no task conflict to reduce and hence this trial type would be unaffected by stimulation. Moreover, since following task conflict reduction, all trial types involving words would have their RTs uniformly reduced, the difference between them would remain the same, and thus the measures of other conflicts will be unaffected.

In contrast, left DLPFC might play a role in resolving just semantic conflict (van Veen and Carter 2005) or resolving both response and semantic conflict (Milham et al. 2001; Parris et al. 2019a, b). Finally, left DLPFC might play little to no role in modifying Stroop conflicts, and thus there will be evidence for no effect on all conflict types.

## Method

### Participants

22 participants (3 male, *M*_age_ = 21.86, SD = 4.04) recruited from the Bournemouth University student population completed the study. One participant withdrew from the study after the first session of DLPFC stimulation after reporting some discomfort. This participant was later replaced. All participants were between 18 and 35 years old, fluent in English and had a normal or corrected-to-normal vision, as well as normal colour vision. Participants were screened for potential risk factors associated with rTMS according to published guidelines (Rossi et al. 2009). Each participant was tested individually across two testing sessions and received course credits or £20 for participating. Written informed consent was obtained from all participants and the study was approved by the Bournemouth University Research Ethics Committee.

### Design

The study used a 2 × 2 × 5 (stimulation condition (DLPFC vs. Vertex) vs. pre/post-stimulation performance vs. trial type) single-blind within-subjects design.

### Stimuli

Twelve unique stimuli were used for each of the five trial types (single-letter string trials, neutral word trials, semantic-associative trials, non-response set trials and incongruent trials). Stimuli were presented individually in uppercase Courier New font, size 42, on a black background. All items were presented in the centre of the screen in one of four colours: red (RGB; 255; 0; 0), blue (RGB: 0; 32; 96), green (RGB: 0; 176; 80), and yellow (RGB: 255; 255; 0), with all words with an association with colour (e.g. red, grass) always being presented in an incongruent colour. The trials consisted of single letter strings: XXX, XXXX, XXXXX, XXXXXX, neutral non-colour words: TOP, CLUB, STAGE, CHIEF; colour-associated words: SKY, TOMATO, LEMON, GRASS; colour words (non-response): PURPLE, GOLD, WHITE, GREY; incongruent colour words: RED, BLUE, GREEN, YELLOW.

### Procedure

Participants were first provided with information about the use of TMS and were given the opportunity to ask questions. After the procedure had been explained and informed consent had been obtained participants completed a computerised Stroop task administered using OpenSesame 3.2 software (Mathôt et al. 2012). Response latencies were recorded using a headset microphone and were measured as the time point a participant started to speak. The experimenter sat behind the participant and recorded errors including incorrect responses (reading the word instead of the colour), corrected responses (starting to read the word, then naming the colour) and noises preceding a response (e.g., coughing or filler words such as ‘um’). Participants were sat approximately 65 cm from the screen which measured 24 inches diagonally. Before beginning the experimental trials, the sensitivity of the microphone in detecting responses was checked in a series of practice trials. The practice trials consisted of 16 colour patches (4 of each response colour: red, blue, green and yellow) presented in a random order. Participants were instructed to name the colours out loud, and each patch remained on the screen until a response had been detected.

After practice, 300 experimental trials were completed. Participants were instructed to respond as quickly and as accurately as possible to the colour of each stimulus whilst ignoring the meaning of the irrelevant word. The stimuli were presented in mini-blocks containing all and only the 12 stimuli for each trial type and the 12 stimuli were presented in random order. Once all 5 trial types had been presented in their mini-blocks, participants would have completed a block (therefore, 60 trials comprised a block). Within each block, each mini-block was presented in a random order. Each trial began with a white fixation dot for 300 ms. The stimuli were then presented and remained on the screen until a response was made or until 3000 ms had elapsed. After a response had been made the stimulus was immediately replaced by a black screen for 1000 ms. After each block of 12 stimuli participants could take a break and pressed the spacebar to start the next block of trials. The task lasted approximately 15 min.

After the initial Stroop task block high-frequency rTMS was performed. All stimulations were performed using a DuoMAG XT stimulator (Rogue Resolutions Ltd, Cardiff, UK) with a figure 8-shaped coil. The EMG was recorded using two pregelled Deymed Diagnostic 22 × 30 mm^2^ Ag–AgCl disposable electrodes placed over the region of the abductor pollicus brevis (APB) belly and associated tendon of the right hand, and a Velcro wraparound Ground Electrode on the right wrist. The resting motor threshold (RMT) of each participant was determined before each stimulation by establishing the lowest setting at which ≥ 5 out of 10 stimulations of the left motor cortex resulted in a minimum MEP amplitude of 50 μV elicited at a given stimulation intensity. Stimulation intensity was set at 110% of the established RMT for each individual. The study used the parameters previously set by Vanderhasselt et al. (2006), with a stimulation frequency of 10Hz and intertrain interval of 26 s. Forty trains were applied in a ca. 20 min period (1,560 pulses per session), with each train lasting 3.9 s. The left DLPFC was defined as the F3 location given by the International 10–20 system (BA8/9; Herwig et al. 2003) and was identified for each participant using the Beam F3 system (Beam et al. 2009). The vertex was identified as the Cz location using the International 10–20 system (Herwig et al. 2003). Immediately post-stimulation, participants completed a further 300 trials of the Stroop task.

The order of the two testing sessions was counterbalanced such that 11 participants received DLPFC stimulation and 11 participants received vertex stimulation on the first session. During the second session, participants completed the same procedure except that they were assigned to the opposite condition (DLPFC or vertex). The two testing sessions were separated by a delay of 1 week and participants were stimulated at the same time of the day. In total, participants completed 1200 trials across the two testing sessions. After completing the experiment participants were fully debriefed and thanked for their time.

### Statistical analysis

Where necessary, we applied the Greenhouse–Geisser correction to ensure the assumption of sphericity. The significance level was set at *p* ≤ 0.05 for all analyses. The data were analysed using a repeated-measures analysis of variance (ANOVA) and significant effects were investigated using paired *t*-tests.

Bayes factors (*B*) were used to assess the strength of evidence for 1-degree of freedom alternative hypotheses, H1, over the null, H0 where results were non-significant. A *B* of above 3 indicates moderate evidence for H1 over H0 and below 0.33 moderate evidence for the H0 over H1. All Bayes factors, *B*, reported here represent the evidence for H1 relative to H0; to find the evidence for H0 relative to H1, take 1/*B*. *B*s between 3 and 0.33 indicate data insensitivity (see Dienes 2014). Here, *B*_H(0, *x*)_ refers to a Bayes factor in which the predictions of H1 were modelled as a half-normal distribution with an SD of *x* (see Dienes 2014, 2016); the half-normal can be used when a theory makes a directional prediction where *x* scales the size of the effect that could be expected. All Bayes factors were calculated with an adjusted standard error where SE = SE × (1 + 20/*df* × *df*) due to the sample size being less than 30 (Dienes 2014). Predictions of the theory were represented as a half-normal scaled with an expected reduction of 26 ms which represents the significant RT reduction in incongruent trial RTs following DLPFC stimulation in Vanderhasselt et al. (2006).

Bayes factors were also calculated for comparisons of conflict types between the vertex and DLPFC conditions. Predictions of the theory were represented as a half-normal scaled with an expected reduction of 55 ms for the overall interference comparison, which represents the average significant interference (incongruent–neutral words) reduction after post-hypnotic suggestion, taken from Augustinova and Ferrand (2012; Experiments 1 and 2) and Zahedi et al. (2019); both these studies, like the present study, employed vocal response Stroop tasks and had an original interference value of ~ 100 ms. Inevitably the expected reduction of the individual components of interference should not be as great and, therefore, we use 1/3 of the prior value for overall interference (which assumes all conflict types contribute equally to interference). Therefore, task, semantic and response conflict are expected to be reduced by 18.33 ms.

## Results

### Analysis of reaction times

Errors rates were low in our experiment and accounted for only 2.86% of all trials. Error trials were removed before running the analyses. Reaction times of correct responses were analysed using a 2 × 2 × 5 (stimulation condition: vertex vs. LDLPFC vs. pre/post vs. trial type: response set incongruent vs. non-response set incongruent vs. semantic associative incongruent vs. non-colour word neutrals vs. repeated letter strings) repeated measures ANOVA. Latencies of correct responses more than two standard deviations away from each participant’s mean in each condition were removed as outliers (Vanderhasslet et al. 2006). This resulted in the exclusion of 4.85% of the trials.

The results of the ANOVA revealed a significant main effect of trial type on reaction times [*F*(1.87,39.26) = 64.95, *p* < 0.001, *η*_p_^2^ = 0.756]. No significant main effect of stimulation condition [*F*(1,21) = 2.58, *p* = 0.123, η_p_^2^ = 0.110] or pre/post [*F*(1,21) = 2.64, *p* = 0.119, *η*_p_^2^ = 0.112] was found.

**Fig. 1:**
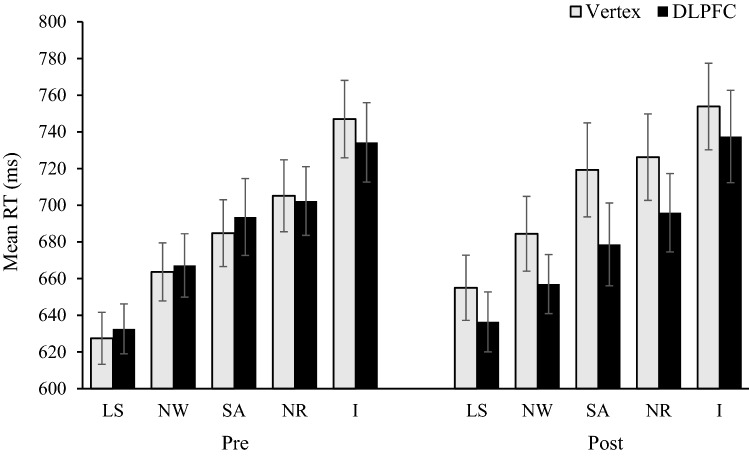
Mean reaction times per trial type for pre and post stimulation as a function of stimulation type. Error bars represent SE. ‘LS’ refers to letter strings. ‘NW’ refers to neutral words. ‘SA’ refers to semantic associates. ‘NR’ refers to non-response set. ‘I’ refers to incongruent

The interaction between stimulation condition and pre/post was shown to be significant [*F*(1,21) = 4.38, *p* = 0.049, *η*_p_^2^ = 0.173] and resulted from an increase in RT from pre- to post-stimulation in the vertex condition. This finding contrasts with those in previous studies in which a pre-to-post decrease in RTs in the DLPFC stimulation condition was reported (Li et al. 2017; Vanderhasselt et al. 2006). The interaction between stimulation condition and trial type [*F*(2.18,45.72) = 0.23, *p* = 0.811, *η*_p_^2^ = 0.011] and the interaction between pre/post and trial type [*F*(4,84) = 0.63, *p* = 0.644, *η*_p_^2^ = 0.029] were non-significant. However, the crucial three-way interaction between stimulation condition, pre/post and trial type was significant [*F*(2.64,55.43) = 3.44, *p* = 0.028, *η*_p_^2^ = 0.141]. Mean reaction times for each condition are displayed in Fig. 1.

This interaction effect was explored using follow-up paired *t* tests. We first compared the difference in baseline (pre-stimulation) performance between the vertex and DPLFC conditions for each trial type. Analysis showed that performance on each trial type before stimulation was not significantly different for the vertex and DLPFC conditions (*p* > 0.261). Bayes factors (two-tailed) for letter string trials *B*_H(0, 26)_ = 0.43, neutral word trials *B*_H(0, 26)_ = 0.42, semantic associative trials *B*_H(0, 26)_ = 0.51, non-response set trials *B*_H(0, 26_ = 0.42, and incongruent trials *B*_H(0, 26)_ = 0.67, were all insensitive but favoured H0.

A comparison of post-stimulation performance revealed a significant reduction in RTs to letter strings [MD = 18.94, SD = 37.90, *t*(21) = 2.34, *p* = 0.029, *d* = 0.23], neutral words [MD = 26.83, SD = 52.48, *t*(21) = 2.40, *p* = 0.026, *d* = 0.28] and semantic associates [MD = 33.01, SD = 54.23, *t*(21) = 2.86, *p* = 0.009, *d* = 0.27] after DLPFC compared to vertex stimulation. The RT reduction on nonresponse set trials was non-significant [MD = 23.00, SD = 52.95, *t*(21) = 2.04, *p* = 0.054, *d* = 0.21] but the Bayes factor indicated evidence for H1, *B*_H(0,26)_ = 3.82. For incongruent trials (9.12 ms) the Bayes factor was insensitive, *B*_H(0, 26)_ = 0.86.

The performance change from pre to post was also compared for each trial type in the DLPFC and vertex stimulation conditions. After vertex stimulation, it was found that RTs to the letter strings [MD = − 27.54,SD = 56.27, *t*(21) = -2.30, *p* = 0.032, *d* = 0.41], neutral words [MD = − 20.79, SD = 45.44, *t*(21) =  − 2.15, *p* = 0.044, *d* = 0.28] and semantic associative trials [MD = − 34.51, SD = 71.30, *t*(21) =  − 2.27, *p* = 0.034, *d* = 0.40] all significantly increased after stimulation. The Bayes factors (two tailed) for non-response set trials *B*_H(0,26)_ = 1.85 and incongruent trials *B*_H(0,26)_ = 0.42 were both insensitive. For the DLPFC condition, there was no significant difference in RTs from pre to post and Bayes factors for letter strings *B*_H(0,26)_ = 0.24 and incongruent trials *B*_H(0,26)_ = 0.22 indicate moderate evidence for H0. The Bayes factors for neutral words *B*_H(0,26)_ = 0.94, sematic associative *B*_H(0,26)_ = 0.68, and non-response set trials *B*_H(0,26)_ = 0.37, were all insensitive but favoured H0.

### Analysis of conflict types

The magnitude of each conflict type was calculated for each participant using the planned comparisons outlined in the introduction. The results were analysed using a 2 × 2 × 4 (stimulation condition vs. pre/post vs. conflict type) repeated measures ANOVA.

The main effect of stimulation condition [*F*(1,21) = 4.08, *p* = 0.056, *η*_p_^2^ = 0.163] and pre/post [*F*(1,21) = 1.84, *p* = 0.190, *η*_p_^2^ = 0.080] were both non-significant. However, there was a significant main effect of conflict type [*F*(3,63) = 60.43, *p* < 0.001, *η*_p_^2^ = 0.742]. The mean magnitude of each conflict type for each condition is displayed in Table 1.

**Table 1:** Mean magnitude of conflict types (ms) for each planned comparison in the pre and post conditions for vertex vs. DLPFC stimulation

Conflict type	Pre		Post	
	Vertex	DLPFC	Vertex	DLPFC
Task conflict (NW-LS)	20.61 (24.09)	32.94 (32.30)	29.44 (29.09)	34.09 (30.25)^a^
Semantic conflict (SA-NW)	21.62 (41.07)	29.51 (35.45)	34.83 (44.39)	28.89 (36.14)
Response conflict (I-NRS)	41.60 (38.49)	34.62 (38.43)	27.60 (36.93)	45.44 (45.33)^a^
Overall interference (I-LS)	101.09 (49.57)	104.10 (52.90)^#^	98.82 (43.88)	122.33 (70.55)^a^

‘SD’ is presented between parentheses. ‘LS’ refers to letter strings. ‘NW’ refers to neutral words. ‘SA’ refers to semantic associates. ‘NRS’ refers to non-response set. ‘I’ refers to incongruent

All conflict effects were significant in all conditions (*p* < .05)

^a^Bayes Factor < 0.3 = Evidence for the null hypothesis of no difference between vertex and DLPFC stimulation

The interaction between stimulation condition and pre/post [*F*(1,21) = 1.06, *p* = 0.314, *η*_p_^2^ = 0.048], the interaction between stimulation condition and conflict type [*F*(2.11,44.48) = 0.61, *p* = 0.557, *η*_p_^2^ = 0.028], and the interaction between pre/post and conflict type [*F*(3,63) = 0.54, *p* = 0.660, *η*_p_^2^ = 0.025] were all non-significant. The three-way interaction between stimulation condition, pre/post and conflict type was also non-significant [*F*(2.34,49.19) = 2.27, *p* = 0.106, *η*_p_^2^ = 0.098].

Comparisons between the critical and baseline trial types (the conflict effects) were significant (*p* < 0.05), providing evidence for the task, semantic and response conflict in all conditions. Since no significant interaction effect was observed the mean differences in the magnitude of conflict types between the vertex and DLPFC conditions were not analysed. However, Bayes factors were calculated for these comparisons to assess evidence for H1 vs. H0. For the pre-stimulation comparisons the Bayes factor (two-tailed) for overall interference provides evidence for no difference, *B*_H(0,55)_ = 0.14. The Bayes factors (two-tailed) for task conflict, *B*_H(0,18.33)_ = 0.85, semantic conflict, *B*_H(0,18.33)_ = 0.60, and response conflict *B*_H(0,18.33)_ = 0.55, were all insensitive but favoured H0.

For the post-stimulation comparison the Bayes factor for overall interference provides strong evidence for no effect of stimulation, *B*_H(0,55)_ = 0.06. The Bayes factors for task conflict, *B*_H(0,18.33)_ = 0.29, and response conflict, *B*_H(0,18.33)_ = 0.29, both suggest moderate evidence for H0. The Bayes factor for semantic conflict, *B*_H(0,18.33)_ = 0.76, was insensitive but favoured H0. Therefore, the results of the Bayesian analysis suggest that stimulation of the left DLPFC does not reduce overall Stroop interference, task conflict or response conflict. Consistent with previous findings then (Friehs et al. 2020; Li et al. 2017; Vanderhasselt et al. 2006) we found no evidence for an effect of stimulation of left DLPFC on interference control. In contrast to previous studies, however, we provide Bayesian evidence for no difference.

### Analysis of errors

The total error rates for each trial type were, 0.74%, 1.53%, 2.80%, 2.80%, and 6.42% for the letter strings, neutral words, semantic associates, nonresponse set, and incongruent trials, respectively. Error rates were subjected to a 2 × 2 × 5 (stimulation condition vs. pre/post vs. trial type) repeated measures ANOVA.

The results of the ANOVA revealed a significant main effect of trial type [*F*(1.95,40.94) = 38.44, *p* < 0.001, *η*_p_^2^ = 0.647] and pre/post [*F*(1,21) = 11.84, *p* = 0.002, *η*_p_^2^ = 0.360]. However, the main effect of the stimulation condition was nonsignificant [*F*(1,21) = 3.00, *p* = 0.098, *η*_p_^2^ = 0.125].

**Fig. 2:**
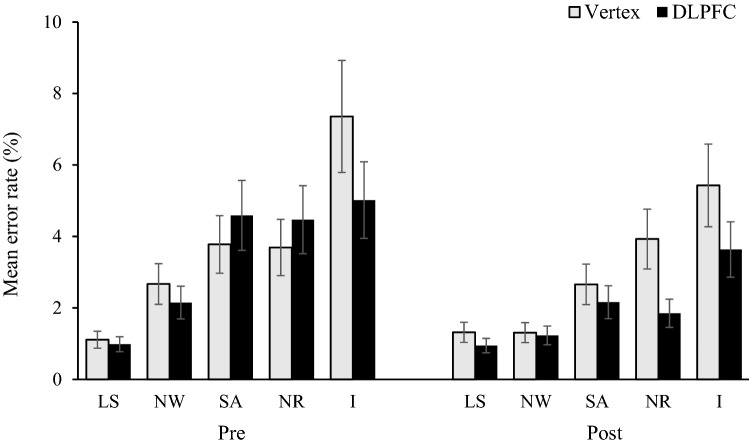
Mean error rates (%) per trial type for pre and post stimulation as a function of stimulation type. Error bars represent SE. ‘LS’ refers to letter strings. ‘NW’ refers to neutral words. ‘SA’ refers to semantic associates. ‘NR’ refers to non-response set. ‘I’ refers to incongruent

The interaction between stimulation condition and pre/post was shown be significant [*F*(1,21) = 5.68, *p* = 0.027, *η*_p_^2^ = 0.213]. However, the interaction between stimulation condition and the trial type was nonsignificant [*F*(1.72,36.16) = 1.10, *p* = 0.336, *η*_p_^2^ = 0.050], as was the interaction between pre/post and trial type [*F*(2.56,53.77) = 2.64, *p* = 0.067, *η*_p_^2^ = 0.112]. The three-way interaction between stimulation condition, pre/post and the trial type was also nonsignificant [*F*(2.60,54.56) = 1.52, *p* = 0.224, *η*_p_^2^ = 0.067]. Error rates for each condition are displayed in Fig. 2.

## Discussion

The aim of the present study was to investigate the effect of left DLPFC stimulation on overall, task, semantic, and response conflict in the Stroop task. Previous fMRI studies and influential neurocognitive models suggest that left DLPFC is involved in conflict resolution (e.g., Banich 2009, 2019; Banich et al. 2019; Botvinick et al. 2001; MacDonald et al. 2000). In contrast to these accounts, previous work employing stimulation of the left DLPFC report no changes to the Stroop effect, but do report reductions in reaction times on congruent and incongruent trials (Friehs et al. 2020; Li et al. 2017; Vanderhasselt et al. 2006). Whilst these studies concluded that enhancing left DLPFC has no effect on conflict control two did not provide evidence for the null (Li et al. 2017; Vanderhasselt et al. 2006), all three employed only sham stimulation as a control condition, and none addressed the effect of stimulation on the underlying components of Stroop interference.

Consistent with previous stimulation findings, the results of the present study show a clear effect of left DLPFC stimulation on task performance; as evidenced by both reduced RTs and reduced errors. However, the pattern in our data were different in that when comparing performance change from pre to post stimulation in the DLPFC condition it was shown that RTs on all trials were non-significantly different whereas in the vertex condition, RTs on all trials increased after stimulation. Therefore, a possible interpretation of our findings is that vertex stimulation resulted in a reduction in performance rather than that DLPFC stimulation improved performance. However, the vertex condition is our baseline condition and permits us to see what happens to RTs when actual stimulation occurs in a putatively non-critical region of the cortex for cognitive control. It is precisely this somatosensory effect that was missing in previous studies and thus one of the important contributions made by the present study. Thus, looking at the baseline condition only, we can argue that rTMS stimulation to a non-critical region results in increased RTs. This we interpret to be due to the general effect of cortical stimulation. Since this increase does not happen in the DLPFC stimulation condition, we interpret the relatively shorter post-stimulation RTs in this condition compared to the post-stimulation vertex condition as representing a decrease in RTs due to the DLPFC stimulation. This improved performance post-DLPFC stimulation was supported in our data by reduced errors.

A further important contribution from the present work is that our data suggest that left DLPFC stimulation does not modify the Stroop interference effect where the null is supported by sensitive Bayes Factors (see also Friehs et al. 2020) and, moreover, further suggests that task, semantic and response conflict are also unaffected where evidence for the null is provided for task and response conflict. Our results, therefore, replicate and extend those from previous stimulation studies and, moreover, are inconsistent with interpretations of neuroimaging data (Milham et al. 2001; Parris et al. 2019a, b) and predictions from leading neurocognitive models of selective attention and cognitive control (Banich 2009, 2019; Botivnick et al. 2001; van Veen & Carter, 2005). As noted recently by Baumert et al. (2020) the absence of conflict modulation does not support the idea that the left DLPFC is causally involved in interference control. However, a caveat to this conclusion is the possibility presented recently by Hartwigsen (2018) who argued that stimulation of a subcomponent region of a neural network might result in a temporary re-organisation and subsequent compensation of function by another region of the network. The process of temporary re-organisation would result in the absence of a modification of the specific cognitive function under test.

A more effective attentional set should result in reduced competition between the target task set of colour naming and the exogenously activated task set of word reading; that is, it would reduce task conflict. To support the hypothesis that stimulation of the left DLPFC reduces task conflict by imposing an enhanced attentional set, it was necessary to show that relative to our baseline letter string trials, RTs to neutral word trials were significantly reduced after stimulation. However, comparisons of post-stimulation performance in the vertex and DLPFC conditions revealed that performance on both letter string and neutral word trials were improved to the same extent resulting in no significant change in the magnitude of task conflict. Thus, whilst previous studies have concluded that a reduction in RTs is consistent with a role for the left DLPFC in setting an attentional set, it is not clear why a modification of the attentional set would not also modify task conflict.

One interpretation of the present data is that facilitation of DLPFC is merely setting a new response threshold; an effect similar perhaps to changes in response threshold on word naming times brought about by mixing high and low-frequency words (Lupker et al. 1997, 2003). In these studies, Lupker and colleagues show that word reading response times can be modified by mixing words that are read quickly (e.g., high-frequency words) with words that are read slowly (e.g., low-frequency words). In all cases, they showed that the more slowly named stimuli were named faster when mixed with word stimuli that had quicker naming times; an effect they attributed to the strategic use of a time-criterion to guide responding. Such an account is not entirely inconsistent with the attentional set account of reduced RTs following stimulation of the left DLPFC if one assumes that the attentional set is not one that biases the target task of colour naming but instead is one that enhances attention to the stimuli and modifies response thresholds. Indeed, the finding of reduced RTs to both incongruent and congruent stimuli in previous studies (Li et al. 2017; Vanderhasselt et al. 2006) is not consistent with an account based on enhanced attention to colour naming since this would reduce the input from the word dimension which would decrease, not increase, facilitation on congruent trials (and thus increase and not decrease RTs to congruent trials). Only a reduction in task conflict could explain reduced RTs to both incongruent and congruent stimuli since a reduction in task conflict would decrease RTs to incongruent trials and permit facilitation to be more fully expressed on congruent trials (Goldfarb and Henik 2007). But as the present data suggest, task conflict is not reduced. Thus, our results suggest that left DLPFC stimulation might play a role in setting response thresholds.

An alternative interpretation of speeding responses when correctly classifying the colour of presented words is that the left DLPFC plays a role in the determination of working memory capacity (Baumert et al. 2020). Baumert et al. argued that enhanced working memory capacity would facilitate responding in the Stroop task, independent of trial type because the correct response keys are kept active in working memory (see also Frings et al. 2018). Such an account is not inconsistent with the aforementioned response threshold account.

Our analysis revealed that RTs on incongruent trials were not significantly reduced after DLPFC stimulation. This finding is somewhat surprising given that previous studies employing similar parameters demonstrate a reduction in RTs on incongruent trials (Li et al. 2017; Vanderhasslet et al. 2006). Nevertheless, in contrast to these studies we maximised response conflict in our experiment by employing a pure block design (Hasshim and Parris 2018) and a vocal response (Augustinova et al. 2019). Thus, our data might indicate that facilitation of the left DLPFC leads to a decrease in RTs except when response conflict is high. If we assume that the reduction in RTs on all other trials reflects the facilitation of an ‘attentional set’, and that this effect can be negated on incongruent trials when response competition is high, then our findings remain somewhat consistent with the role of the left DLPFC in implementing top-down attentional control supported in previous TMS studies (Li et al. 2017; Vanderhasselt et al. 2006) and proposed by influential findings and models of cognitive control (Banich 2009, 2019; Botvinick et al. 2001; MacDonald et al. 2000).

It has previously been suggested that while the left DLPFC is responsible for actively preparing for a specific task in the presence of a distracting task (Vanderhasselt et al. 2010), the right DLPFC is recruited after conflict detection to subsequently minimize conflict (Vanderhasselt et al. 2009; see Friehs et al. 2020 for a discussion of hemispheric lateralisation of control in the Stroop task). According to Vanderhasselt et al. (2009) left DLPFC activation is related to a preparatory and temporary increased attentional set, whereas the right DLPFC is related to large-scale cognitive control adjustments in a conflict-driven context which enables us to build representations that bias behaviour towards an appropriate response. In an fMRI study employing neutral, non-response set and incongruent trials Milham et al. (2001) reported that only response conflict (indexed using an incongruent–non-response set comparison) elicited activation in the right PFC, whereas the left PFC was activated by conflict at the response and pre-response levels. This suggests that while both hemispheres of the prefrontal cortex are recruited by the cognitive demands of the Stroop task, their involvement may differ depending on the type of conflict that arises. However, these findings are the subject of continual debate (Chen et al. 2013; Song and Hakoda 2015; van Veen and Carter 2005) and neither the present results nor those from recent research using optimised measures of response and semantic conflict are consistent with this notion (Parris et al. 2019a, b).

An interesting direction for future research would be to examine the effect of right DLPFC stimulation on levels of the various types of conflict in the Stroop task given the potential for lateralisation of control raised by the work of Milham et al. and recent research showing an effect of right DLPFC stimulation on congruency sequence effects (Friehs et al. 2020). Given our use of blocked presentation of trial types to optimise measurement of conflict types (Hasshim and Parris 2018), we are unable to measure congruency sequence effects, but this potential lateralisation of the function should be investigated further. Moreover, Friehs et al. also employed online stimulation whilst the present study and most previous studies used offline stimulation; a difference that could prove to be important in producing the reported effects.

We employed vertex stimulation as a control condition since numerous previous studies have suggested that this region is unrelated to task performance (e.g., Boschin et al. 2017; Hayward et al. 2004), and a recent fMRI study investigating BOLD signal changes across the whole brain linked to vertex stimulation, provides support for its use as a control site (Jung et al. 2016). However, Jung et al. (2016) did show that low-frequency rTMS of the vertex resulted in the deactivation of regions within the default mode network (DMN) suggesting that vertex stimulation is not entirely inert. While we assume that vertex stimulation does not result in any functional changes that effect task performance, more research is needed to fully understand the impact of vertex stimulation at different TMS parameters.

While we employ vertex stimulation to better control for the somatosensory effects of TMS, the precise neural effects of such stimulation at different TMS parameters are relatively unknown. We assume that vertex stimulation produces no neural effects that influence task performance and as such explain the significant difference between conditions at post-test as being due to a general worsening in performance due to stimulation which is counteracted by the beneficial effects of DLPFC stimulation. However, we are unable to conclusively rule out the possibility that vertex stimulation impairs performance as a result of cortical effects. Furthermore, in our experiment the Beam F3^2^ and International 10–20 system were used to define the left DLPFC (F3) and vertex (Cz). Although this method provides an acceptably approximate localization method and has been shown to be better than the 5.5 cm method (Trapp et al. 2020), MRI-based neuronavigation is more accurate (Mir-Moghtadaei et al. 2015). Finally, it should be noted that whilst high frequency (≥ 5-Hz) repetitive TMS (rTMS) is typically considered to have an excitatory effect on underlying neurons (Pell et al. 2011), this is accurate for motor cortex stimulation, and cannot be assumed to be true for nonmotor regions (Bergmann and Hartwigsen 2020). Neuroimaging is considered crucial to provide strong proof of target engagement.

## Conclusion

In sum, our findings suggest that left DLPFC has no causal role in modifying overall, task nor response conflict (our data were insensitive for semantic conflict, although the Bayes factor favoured the null). Importantly, we also show for the first time that stimulation of the DLPFC enhances performance compared to an active stimulation control condition (cf. Li et al. 2017; Vanderhasselt et al. 2006, 2007). We have argued that the failure to observe a reduction in task conflict is problematic for the previously proffered attentional set account. Instead alternative accounts of the role of the left DLPFC in Stroop task performance were suggested according to which the left DLPFC either implements an attentional set that facilitates faster responses by modifying response thresholds or facilitates responding in the Stroop task by keeping the correct response keys active in working memory (Baumert et al. 2020; Frings et al. 2018). However, in contrast to previous studies our data suggest that the effect of left DLPFC stimulation may be limited to situations where response competition is small. In instances where the expectancy of response conflict is high, the right DLPFC may be recruited to a greater extent to implement permanent macro-adjustments in cognitive control (Friehs et al. 2020; Kerns et al. 2004; Milham et al. 2001; Vanderhasselt et al. 2007, 2009).

## Data Availability

The data and materials are available at https://osf.io/ehp84/?view_only=8bdb243ca41e468295bedfefdb0c8815.

## Footnotes

1 The Beam F3 method was created as a way of reducing human error and time consumption when localising F3. The numerous measurements normally required take time and are prone to error. Beam F3 system involves taking only three skull measurements (distance from tragus to tragus, distance from nasion to inion, head circumference) which are inputted into the F3 locator program. The program then produces two values that are used to locate F3.

2 The Beam F3 method was created as a way of reducing human error and time consumption when localising F3. The numerous measurements normally required take time and are prone to error. Beam F3 system involves taking only three skull measurements (distance from tragus to tragus, distance from nasion to inion, head circumference) which are inputted into the F3 locator program. The program then produces two values that are used to locate F3.
